# Harmonizing the Interests of Free Speech, Obscenity, and Child Pornography in Cyberspace: The New Roles of Parents, Technology, and Legislation for Internet Safety

**DOI:** 10.1100/tsw.2009.147

**Published:** 2009-11-18

**Authors:** Amos O. Olagunju

**Affiliations:** Department of Statistics and Computer Networking, St. Cloud State University, St. Cloud, USA

**Keywords:** Internet safety, child pornography, obscene material, technological security device, educational material

## Abstract

Inadvertent access to website addresses and spam e-mails continue to make pornography rampant on the Internet in schools, homes, and libraries. Collectively, parents, teachers, and members of the community must become more aware of the risks and consequences of open access to the Internet, and the distinction between censorship and Internet access filtering. Parental involvement is crucial for raising children with healthy Internet habits to access social and educational materials. Although generations have coped with different times and trials, technology is ushering in new trials. Parents and communities cannot ignore the present and future technology ingrained into the lives of children. This paper contends that parents armed with legislation and technological security devices for access to the Internet ought to strengthen the character of online Internet safety. The discussion is focused on the roles that parents, communities, technology, and laws should play in order to protect children from obscene and pornographic threats from cyberspace. It is argued that the roles of education and technology should outweigh the legislative interventions of governments. A critique of significant litigations and laws on obscenity and pornography is presented. The paper offers a variety of security tools and techniques for protecting children from Internet access to obscene and pornographic materials. The impacts of pornographic materials on the welfare of children, adolescents, women, and families are discussed.

## INTRODUCTION

Today, pornography is one of the most contentious subjects of debate despite deliberate efforts by law enforcement agencies and governments around the world to limit the accessible pornographic materials on the Internet. Child pornography is a crime all over the U.S. In the U.S., child pornography is legally defined as the visual portrayal of sexually overt demeanor by drawings, cartoons, sculptures, paintings, photographs, films, videos, and computer-generated images. In American courts, the definition of obscenity has been difficult to ascertain. Obscenity is any conduct, manifestation, or phrase that defies the norms of sexual morality. Child pornography is obscene when it illustrates a minor engaged in sexually open behavior. Child pornography is on the rise because of the rapid access and use of the Internet by youngsters. There are real live photos of children and software that are used to generate offensive animated pornographic images of children on the Internet. The recurrently convalescing image-enhancing software makes it easier to create and share pornographic materials worldwide via the Internet.

According to the U.S. National Center for Missing and Exploited Children[39], about 20% of youngsters age 10–17 who frequently use the Internet have been sexually importuned online, and about 25% were reluctantly exposed to materials of naked people and sexual engagements. Children erroneously access the Internet pornography through inaccurately directed searches of stealth websites, deceptive universal resource locators, childlike word searches (such as toys, dogs, Britney Spears, Disney, Pokémon, Barbie, and Nintendo), spontaneous spam e-mails, and real-time instant message links with sexual predators[44]. Approximately 25% of pornography sites abuse trendy brand names of children's materials to confuse the Internet search engines. Unfortunately, participation in antisocial sexual behaviors, particularly rape later in life, is associated with the exposure of youngsters under the age of 14 to pornography. In fact, as much as 53% of child molesters have viewed pornography. Consequently, viewing pornography can rapidly make adolescents act out sexually against younger, smaller children[44].

Freedom of speech is the right to express an opinion in public freely without suppression or moderation by the government. The right to freedom of speech is guaranteed under the international law through various human rights documents under Article 19 of the Universal Declaration of Human Rights[64], and Article 10 of the European Convention on Human Rights[16] guarantees the right to free speech. The implementation of freedom of expression is lacking in many countries around the world. In reality, the right to free speech is not absolute and degree of freedom varies vastly around the world. The industrialized countries use different approaches to harmonize freedom with order. The First Amendment to the U.S. Constitution[11] prohibits the Federal Legislature from enacting laws infringing on freedom of speech. The First Amendment hypothetically grants unconditional freedom since it lays the yoke upon the state to exhibit when (if) a restraint of freedom is obligatory. Unfortunately, the principle that restraints are the exceptions and free expression the rule are not fulfilled in most liberal democratic systems. There are conflicts in the interpretations of cyberspace democracy, cyberspace hegemony, and freedom of speech in cyberspace[20]. Should obscene and pornographic materials continued to be targeted towards children on the Internet because of democracy and freedom of speech in cyberspace? This paper provides insights into the educational roles of parents and the impact of technology on protecting children on the Internet.

## NOTEWORTHY U.S. CHILD PROTECTION LAWS

The Children's Online Privacy Protection Act of 1998 (COPPA), enacted in April 2000, shelters the personal information of adolescents under age 13 in chat rooms, on bulletin boards, in instant messaging, and from Web operators. The Online Privacy Protection Act of 2003 protects the personal information of persons above 13 years not covered by COPPA.

The Communications Decency Act (CDA) of 1996 is the first legislative effort that protects children from Internet pornography. The U.S. Supreme Court delivered a blow on the CDA's indecency provisions in the *Reno v. ACLU* case[47], when it found that the anti-indecency provisions infringed the freedom of speech provisions of the First Amendment.

The Children's Internet Protection Act of 2000 (CIPA) obligates public schools and libraries to use a portion of the Federal funds they receive to filter Internet access. Claiming that it violated the First Amendment, the American Civil Liberties Union, the Center for Democracy and Technology, and the

American Library Association challenged the CIPA in court. In 2003, the U.S. Supreme Court overturned a Philadelphia judicial ruling that declared CIPA as unconstitutional.

The Cybermolester Enforcement Act of 2003 introduced the minimum mandatory sentences for Internet child molesters.

In 2002, the U.S. Supreme Court upended the Child Pornography Prevention Act of 1996 (CPPA) by a 6-3 ruling. Justices Kennedy, Stevens, Souter, Bader, Breyer, and Thomas concurred that the bill was overexpansive in prohibiting computer-generated images of children engaging in sexual activities.

The Protection Act of 2003, known as the AMBER Bill (America's Missing: Broadcast Emergency Response), was passed by the House and Senate and signed by President Bush. The bill prohibits virtual child pornography, and allows Federal law enforcement agencies to use a nationwide emergency system to alert the public about missing children, and wiretapping and electronic surveillance in the investigation of child pornography. The U.S. Department of Justice Child Exploitation and Obscenity Section has established a special forensics unit that investigates illegal Internet obscenities in order to collect evidence in support of prosecuting child exploitation and adult obscenity cases.

## STRIKING PORNOGRAPHY COURT CASES

The U.S. Federal government and states have permission to confine obscenity or pornography. Obscenity usually has no protection under the First Amendment and pornography is subject to modest regulation. However, the precise definitions of obscenity and pornography have been altered over time.

The U.S. Supreme Court endorsed the same obscenity yardstick used in a renowned British case, *Regina v. Hicklin*, LR 3 QB 360 (1868)[46], in its *Rosen v. United States*, 161 U.S. 29, 40 (1896)[48], decision in 1896. According to the Hicklin benchmark, a material is obscene if it is likely "*to deprave or corrupt those whose minds are open to such immoral influences, and into whose hands a publication of this sort may fall.*" Unfortunately, the U.S. declared the Hicklin test unsuitable in 1957 in its ruling in *Roth v. United States* 354 U.S. 476 (1957)[49]. The Roth test for obscenity was:

> *Whether to the average person, applying contemporary community standards, the dominant theme of the material, taken as a whole, appeals to the prurient interest*[49].

In 1973, the Court extended the Roth test in its *Miller v. California*, 413 U.S. 15 (1973), case decision[34]. A material is obscene under the Miller Test if, using modern-day community measures, it could be alluring to the prurient curiosity by a typical person and it portrays sexual behavior in a boldly distasteful way without any serious artistic, literary, political, or scientific value. Thus, a material considered obscene in a community may not be considered obscene in another neighborhood because there are no national standards for gauging the values of materials. Child pornography is not subject to the Miller test, as the Supreme Court decided in 1982. The Court felt that the government's interest in protecting children from abuse was paramount.

In 1964, Justice Potter Stewart, in the case of *Jacobellis v. Ohio*, 378 U.S. 184 (1964), even though he could not precisely define pornography, notably stated that, "*I know it when I see it*"[26]. However, personal possession of obscene materials at home may not be prohibited by law. Writing for the Court in *Stanley v. Georgia*, 394 U.S. 557 (1969), Justice Thurgood Marshall wrote:

> *If the First Amendment means anything, it means that a State has no business telling a man sitting in his own house what books he may read or what films he may watch*[54].

Although obscene materials may be viewed only in private, it is not unconstitutional for the government to prevent the mailing or sale of indecent items. The Child Pornography Prevention Act (CPPA) of 1996 "*prohibited child pornography that does not depict an actual child.*" The *Ashcroft v. Free Speech Coalition*, 535 U.S. 234 (2002), case argued and invalidated the CPPA because it was exceedingly broad and unconstitutional under the First Amendment. In the verdict, Justice Anthony M. Kennedy wrote:

> *First Amendment freedoms are most in danger when the government seeks to control thought or to justify its laws for that impermissible end. The right to think is the beginning of freedom, and speech must be protected from the government because speech is the beginning of thought*[3].

Over the years, the U.S. courts have upheld certain regulations banning pornography as a way to protect children; for instance, the zoning regulation limits where pornographic materials can be viewed.

## ALARMING INTERNET CHILD PORNOGRAPY STATISTICS

The U.S. pornography revenue was $13.3 billion in 2006; worldwide $97.06 billion[23]. $3,075.64 was spent on pornography, 28,258 Internet users were looking at pornography, 372 Internet users were using search engines to look for adult terms every second and a new pornographic video was produced every 39 minutes in the U.S. in 2006. One in four Internet search requests is connected to pornography[28].

The Top Ten Adult Search Requests were “sex”, “adult dating”, “adult DVD”, “porn”, “sex toys”, “teen sex”, “free sex”, “adult sex”, “group sex”, and “free porn” in 2006. Twelve percent of all websites were pornographic websites. There were 4.2 million pornographic websites, 420 million pornographic web pages, and 68 million daily pornographic search engine requests (or 25% of total search engine requests). Seventy-nine percent of unwanted exposure of pornography to youths occurred in the home[43]. The largest group of viewers of Internet pornography was children between the ages of 12 and 17[17].

Child pornography is one of the fastest growing businesses online. In 2004, the Internet Watch Foundation located 3,433 child abuse domains; in 2006, the child abuse domains increased significantly to 10,656[24]. Of all known child abuse domains, 54% were housed in the U.S.[24].The 13,585 hardcore pornographic video/DVD titles released in the U.S. in 2005 represent a dramatic increase over the 1,300 titles in 1988[28].

The fastest growing demand in commercial websites for child abuse is for images depicting the worst type of abuse, including penetrative sexual activity involving children and adults, and sadism or penetration by an animal[24]. Gnutella had 116,000 daily “child pornography” requests in 2006[23]. About 30% of Internet pornography customers are female[28,42].

In a study of arrested child pornography possessors, 40% had both sexually victimized children and were in possession of child pornography. Of those arrested between 2000 and 2001, 83% had images involving children between the ages of 6 and 12; 39% had images of children between the ages of 3 and 5; and 19% had images of infants and toddlers under the age of 3[40].

The Internet had a significant impact on divorces in 2002. Divorce cases involved couples who spent too much time in sexual forum chat rooms and had compulsive interests in pornographic websites[12].

Pornography promotes sex trafficking, and traffickers carry filming equipment and cameras for creating and selling pornographic materials. Each year, 600,000–800,000 women, men, and children are trafficked internationally, and 2–4 million are trafficked within countries. Women account for 80% of humans trafficked, of which 70% is for sexual purposes such as prostitution[31]. It is not surprising then that in the U.S., one in four adolescent females (ages 14–19) is infected with at least one sexually transmitted disease, and 15% have more than one sexually transmitted disease[1].

According to a National Children’s Homes report, the number of Internet child pornography images increased 1500% from 1988 to 2003. Approximately 20% of all Internet pornography involved children, and more than 20,000 images of child pornography were posted on the Internet every week[41]. Child pornography has become a $3 billion annual industry[57]. The alarming statistics reveal that the sex industry and pornography are becoming more prevalent in North American culture and society.

## BACKGROUND AND RELATED STUDIES

Parents are ill equipped and unable to teach children about safety in cyberspace[15,21]. The U.S. government has been concerned about the letdown of the Web filtering technology to protect children from obscene and pornographic materials[6]. The failed efforts to enact the Child Online Protection Act compelled the U.S. Department of Justice to issue subpoenas to various Web search engine providers[30,33]. The purpose was to obtain Web addresses and records of searches to serve as evidence that the filtering technology could protect children from inappropriate materials on the Internet. All but the Google Internet search providers turned search records over to the U.S. government[18]. Is this an issue of the constitutional rights of the Internet search engine providers to protect information? What about the legal protection of children from viewing inappropriate materials on the Internet?

What measures should be taken to protect youngsters from obscene and pornographic materials on the Internet? Kimberly Mitchell and Michele Ybarra conducted research on the online behaviors of youths who engage in self-harm and found that technology has become an integral part of their daily lives. Although the research performed by Mitchell and Ybarra is specific to a group who deliberately self-harm, the results revealed Internet usage concerns of interest to educators and parents. Of particular interest is the assertion by Mitchell and Ybarra that:

> *Although empirical research is practically non-existent, recent reports, based on investigations of online chat rooms, message boards, and newsgroups suggest that the Internet can have both beneficial and detrimental influences on youth who are engaging in deliberate self-harm*[37].

This argument could be extended to include the benefits and potential unfavorable influences of the Internet access on all unsupervised children. Mitchell and Ybarra also emphasize that:

> *It is an important wake-up call however for adolescent health professionals to be mindful of including online behaviours in the risk assessments*[37].

Indeed, this is a wake-up call for parents, educators, and communities to begin monitoring what the youths and children see and do online.

Sara Bushong, in her article "Parenting the Internet"[4], cited the primary reason for purchasing family computers as the need to enhance the education of the children. Unfortunately, most parents do not know how to monitor the online computer activities of their children. Bushong reported that in a suburban community in Ohio, none of the research participants from various families was aware that local library catalogues and electronic databases were reachable from home computers.

Consequently, parents today require a better understanding of how the Internet could be used to access learning resources devoid of obscene and pornographic materials in order to compliment the education of the children. Bushong advocated the need for parental involvement in combating online access to obscene and pornographic materials by children when she called on parents to:

> *Place the Internet-accessible computer in a visible, high-traffic living space in the home*[4].

This call serves as a reminder to children that materials viewed on the Internet are accessible to other users, and it also emphasizes that parents need to teach children about Internet access safety. Bushong reiterated that parents should:

> *Search online with children, discussing content, web site evaluation, advertising practices, privacy policies, personal information and family rules*[4].

Moreover, Matthew Eastin, Bradley Greenberg, and Linda Hofschire, in a Kaiser Family Foundation study, cited computer and Internet use as becoming increasingly accessible to children from an alcove, the bedroom. In addition, Internet usage competes with traditional activities, such playing outside, in the lives of children today[15].

Eastin, Greenberg, and Hofschire studied the impacts of parenting styles on Internet usage. The authoritarian, authoritative, permissive, and neglectful styles of parenting were evaluated. The authors reported that:

> *Parenting style has a significant effect on almost all mediation techniques investigated. Authoritative parents use evaluative and restrictive mediation techniques more than authoritarian and neglectful parents. Regardless of parenting style, parents whose teenagers have access to the Internet in their bedroom spend more time online. In fact, time online almost doubles with access in the bedroom*[15].

Valcke et al. surveyed a group of 1700 primary school headmasters and pupils in 4$^{th}$, 5$^{th}$, and 6$^{th}$ grades at 78 schools in Flanders about their use of the Internet. The focus of the research was to gain insights into the perceptions of safe use of the Internet. The authors emphasized the importance of parental involvement in the discussion of inappropriate Internet content with the children. Unfortunately, the study revealed that out of 1626 pupils, 52% said they were never or were hardly ever monitored when using the Internet at home. The authors concluded that parents who teach children online safety have a significant impact on reinforcing programs designed to protect youngsters from Internet threats such as pornography[58]. Undeniably, children are sometimes exposed to Internet pornography through inadvertent website addresses and spam e-mails.

Rich Chapin provided unique perspectives into the nature of the cyberspace threats to children and offered possible solutions. According to Chapin:

> *It is both ubiquitous and unavoidable that students may access the Internet from school, at home, from the library, and with friends. We should understand the difference between censorship and filtering. We monitor their playmates and don't let them talk to strangers. This is to a very great degree what good parenting is all about. Many schools are adopting "Acceptable Use Policies" –documents that describe how the Internet is to be used in school and the associated consequences for its misuse. Schools should post their policies on their Web sites, send them home to parents and teach them to students*[6].

Thus, parents, teachers, and community members collectively must be aware of the risks and consequences associated with open areas where children explore resources on the Internet. Children require protection from accidental access to obscene and pornographic materials on the Internet. In a proactive step at home, parents should have and review a document of the Internet access policy for their children, and post it near the computer. Schools should also assist parents to implement Internet access filtering software and policies at home. Moreover, the alliance of parents and schools would provide a better defense against Internet threats for the children.

Steve Dorman, a proponent of parental and teacher involvement, offered five proactive recommendations for use by parents and teachers in promoting Internet safety:

> *Children using the Internet should be supervised in the same way they are when viewing television.*
>
> *Parents, teachers, and schools may purchase a filtering device, which can filter many pages that parents and teachers might consider harmful or offensive.*

*Children should avoid bulletin boards and chat rooms, where they are introduced to strangers.*

*Children should be taught how to use the Internet to find the information they need and how to avoid useless and harmful sites.*

*Teachers should follow school acceptable-use policies and procedures developed for student and faculty use of the Internet*[14].

Without a doubt, there ought to be an analogy between the supervision of television viewing and Internet usage. In fact, parents teach children their address and phone number, and to stay away from strangers. They monitor what children watch on TV and keep track of their children's friends. Now parents ought to assume a more proactive role in teaching and monitoring the Internet online activities of their children.

Rich Chapin recommended consistent involvement of parents and schools in proactive monitoring and reading of the log entries of Web usage by children. Chapin forewarned parents about the limitation of reactive approach to Internet access:

*Monitoring alone can only be reactive, identifying problems after they happen. For proactive control, additional content filtering technology is necessary*[6].

Certainly, there are different tools for monitoring Web usage. There are Web browser tools for monitoring and protecting children from online Internet threats. For instance, the history tool stores all accessed Web pages until the folder is cleared. Proxy servers also provide the history of cached pages. Today, there are sophisticated filtering technologies and reporting tools in proxy servers. The list-based filtering technology is capable of blocking known useless Web sites and domains that contain obscene and sexual content. The filtering technology is a difficult approach to maintain because the rapid growth of the Internet requires constant updating of the list of obscene and pornographic websites. The text filter technology provides a better option for monitoring the activities on websites. The accessed Web contents can be monitored by filtering on specific text items. Unfortunately, the use of text filters to filter out inappropriate words could result in many false-positive websites, such as the denial of users from accessing many legitimate medical websites. Content recognition is the latest filtering technology that employs the concept of artificial intelligence, such as neural network used to provide a more comprehensive content screening and to reduce human dependency on reviewing Web contents.

It is extremely important for parents to be proactively involved in teaching children safety rules in cyberspace. Children need education about the dangers of e-mailing and providing personal information to strangers on the Internet. Companies often market products to children on the Internet. According to Steve Dorman:

*Many sites on the Web exist solely for marketing a product… For example, Dr. Pepper and NBC created a teen tip 'contest' Web page that asked teens to submit their name, age, gender, address, and e-mail address with a teen tip*[14].

The negative impact of this campaign is the lack of a cautionary statement on the website. Moreover, the owners of websites do not seek permission to collect personal information of children from the parents. Parents need to know that:

*The new technology of the Internet may have changed the pace of the problem, but it has not created the problem*[6].

Consequently, parents and teachers must assume the responsibilities of educating the children about the dangers and threats in cyberspace.

## CONSEQUENTIAL IMPACTS OF PORNOGRAPHY ON CHILDREN AND FAMILY

The debate on pornography focused long ago on consumers vs. victims, legal vs. feminist vs. moral views, and censorship vs. freedom of speech prior to the dawn of the Internet[55]. Modern patterns of the production and consumption of pornographic materials via the Internet make children and adolescents more vulnerable to online sexual predators than ever before. Pornography is shifting the cultural values of societies around the world in surmountable and socially costly ways. The propinquity of the sex industry to the private and public arenas is a troubling-fangled incident with the availability of cable and Internet connections for access to a wide variety of obscene sexual materials unprotected by the First Amendment. Thus, the easily accessible pornographic materials via the Internet today require a new debate about their impact on children, adolescents, genders, and people of all ages and socioeconomic groups.

The exponential growth of the fabrication and use of pornographic materials, and their impact on compulsive sexual behaviors, are attributable to the escalating use of the Internet[10,51,52]. The Internet, since its inception, has had significant impact on sexuality and sexual revolution[8]. Pornography and online sexual hobbies are "… a hidden public health hazard exploding, in part because very few are recognizing it as such or taking it seriously"[9].

The Internet pornographic materials have devastating effects on couples and families. The ease of access to inexpensive and secret pornographic materials via the Internet has negative consequences on human sexuality[7,9,29,51,52]. The 17.5% or over 246 million of the Internet users worldwide reside in North America[25]. The Internet is available to over 73% of the North American population[25]. Nearly 42.7% of the Internet users are viewing pornography online[25]. These statistics reveal the disturbing influences and implications of pornographic materials on the social, emotional, spiritual, physical, and intellectual lives of children, women, and men.

The pornography industry presently does not discriminate against young consumers. Sexually explicit materials on the Internet are extremely meddling and can be accidentally happened upon when opening e-mails or searching the Web[36]. A significant 34% of adolescents have been exposed to useless sexual content online[65], a figure that significantly rose by 9% over 5 years[13]. Youths nationwide in the U.S. reported unwanted sexual solicitation and harassment online[35]. Youths at least somewhat often accidentally run into pornography online. These statistics have long-term impacts on the increasing numbers of youths exposed to immature and hazardous sexual pornographic content that impede safety and healthy behaviors[13].

Internet filters are valuable tools for protecting children from sexually explicit content in cyberspace. Unfortunately, visual teasers are displayed on the homepages of nearly 75% of pornographic websites prior to any effort to validate the legal age of users; barely 3% of pornographic websites require proof of age for access to adult content materials; and two-thirds of pornographic websites have no warnings for any sexually explicit materials[56]. The implementation of reliable and effective age-verification systems for protecting children from the pornographic websites remains an open problem. Moreover, there are presently no filtering systems for cell phones with Internet access or iPods capable of transmitting "podnography", notwithstanding their upward reputation with adolescents[13]. Pornography negatively impacts the development of the minds of adolescents as it induces upset and distress due to youngsters' inability to psychologically process obscene images[32,35,36,59,62]. Youths have described their feelings from exposure to online sexual materials as "sick", "yuck", "disgusted", "repulsed", and "upset"[19]. Premature exposure to pornography often results in long-term and frequently depressive feelings[5].

Pornography negatively influences the attitudes and behaviors of adolescents. Adolescents are more likely to perceive women as sex objects after exposure to sexualized media environments[45,60,61]. An investigation of the impacts of frequent exposures of adolescents and young adults to sexually explicit materials revealed (a) normalized unpleasant response to disgusting materials, (b) more forbearance to sexually explicit materials that require additional new materials to sustain interests or arousal, (c) reduced trust of close friends, (d) less yearning of exclusive sex with a partner, (e) negative perceptions of family and raising children, and (f) approval of undiscriminating sexual behaviors as the norm[67]. Moreover, young women exposed to pornographic materials have more odds of developing negative body images[53].

Pornography influences the way the adolescents structure sexuality and their expectations from intimate relationships. Exposure to sexually explicit materials can negatively impact the sexual, social, physical, spiritual, psychological, and emotional development of youngsters and women[28]. Pornographic images have a traumatic impact on the brain, and the psychological welfare of children and adolescents. Young viewers of pornography are more likely to engage in sexual intercourse at an earlier age than peers unexposed to pornographic materials.

There is a strong connection between abuse in marriage and the use of pornography[22,25,50,63]. Wives of pornography users are often solicited to perform pornographic scenes, exploit pornography with their partners, or are forced into prickly or humiliating sexual acts[22,27,50]. It is sad that:

> *Women's experiences of pornography are dismissed as anecdotal, non-representative, and unimportant. Falling into the measurement trap, we have turned to science, instead of to women, for answers…while we debate it, many are coping with its unwelcome presence in their lives*[22].

The role of women in the rising influence of the production and utilization of pornography deserves more attention in order to promote healthy sexuality, long life relationships, and respect for the future generation of children, adolescents, and families.

## DISCUSSION AND RECOMMENDATIONS

Children have access to the Internet from homes, libraries, schools, cybercafes, hotels, churches, and other public and private sources. Youngsters who access pornography on the Internet often hurriedly shift or minimize the computer screen as an adult enters the room, hide compact or digital-video disks, erase the daily Internet history file, spend too much time on the computer at home, library, or school, and are secret or evasive about instant messages with friends.

The behavior of children on the Internet might be positively or negatively influenced by friends, strangers, teachers, parents, family, and community members. Hence, alternative sources of security and roles are required to protect the children from obscene and pornographic materials in cyberspace. In particular, the educational role of parents and the security role of technologies must be aligned with the role of legislation.

Herein are the roles of the parents, communities, and technologies in protecting children from obscenity and child pornography in cyberspace.

It is the responsibility of every parent to protect children from online threats by creating a list of recommended and agreed upon websites and Internet search engines. A parent ought to be knowledgeable of the hardware devices and software tools available for monitoring, tracking, and logging inbound and outbound Internet communications. Every parent should learn the use of software and hardware tools for filtering and blocking unapproved sites, keywords, and phrases that set off actions and pornographic materials. Parents ought to learn to setup individual accounts with restricted Internet access on home computers. Every parent ought to learn the use of online role play in order to teach their children Internet safety awareness[66]. More specifically, it is the role of parents to:

- Set and adhere to rules for children's access to the Internet inside and outside the home
- Educate their children on the use of the Internet as an opportunity and not a legal right

- Inform children never to give out personal information over the Internet without checking with you the parent
- Build confidence and trust in children by using computers for online activities jointly with youngsters
- Deny children from creating online profiles and placing personal photos in MySpace, FaceBook, etc.
- Alert schools to refrain from identifying projects by children's names and pictures on websites
- Report all suspicious or criminal Internet pornographic activities to local and national law enforcement agencies
- Install computers in intense traffic areas at home and not in children's rooms
- Check the Internet access history files, CDs, DVDs often
- Subscribe to a prefiltered Internet Service Provider
- Use security tools to shield children from the IP addresses and instant messages of sexual predators, strangers, and pornographic materials

The U.S. National Research Council, the Computer Science and Telecommunications Board, and other agencies have invested in technological tools and strategies capable of shielding youngsters from Internet pornography[56]. The technological tools proposed in this paper are well aligned with the ongoing outlined industrial technological tools and solutions for securing youngsters from Internet pornography.

As strong forces, communities have the right to regulate or eradicate pornography. City bylaws require limitations on who can buy and sell pornography. The American Center for Law and Justice[2] affirms that citizens and communities may:

- Apply their First Amendment rights to free speech and gathering to protest peacefully, to picket against, and to boycott pornography merchants and stores
- Contact city attorneys for assistance with existing unenforced pornography laws
- Contact city council members about drafting laws if no pornography ordinances exist
- Advocate for pornography merchants to be zoned into specific areas inaccessible to children

Schools and churches should install Internet programs that monitor and record the use of websites by children and members. Website visit histories should be stored at secured locations and must not be erasable. Members must designate accountability persons who receive a weekly, biweekly, or monthly log of all sites each member has visited. The goal is that members will never be able to hide under any cover of anonymity.

The U.S. Congress provides funds for the National Center for Missing and Exploited Children to work jointly with the FBI, U.S. Customs, the U.S. Postal Inspection Service, and state and local law enforcement agencies in Internet Crimes Against Children Task Forces. The Morality in Media sponsors a website[38] for reporting online child pornography or exploitation, or unsolicited pornographic adult e-mails. A former FBI agent reviews and forwards pornography reports to the U.S. Attorney of the appropriate state. Prosecuting the Internet pornography crimes remains a major challenge in the U.S. because the perpetrator and victim often live in different states. It is the responsibility of all citizens to report all Internet pornography crimes.

Today, there are tools for blocking downloads, spyware, peer-to-peer communicating, online chatting, video, and proxy server. There are complete virus protection and antispam software. There are tools for reporting Web usage, authenticating websites and managing bandwidth. There are firewalls and context-sensitive pornography filters. Unfortunately, the majority of these technological tools require steep learning curves for parents. The new challenge for the information technologists consists of developing user-friendly, self-paced learning tools for parents, while simultaneously building intelligent self-administering cyberspace tools.

## CONCLUSION

The Internet provides access to a vast array of tools for adults to manage personal finances, bank online, manage investments, pay bills, monitor news, shop online, listen to music, watch movies, send e-mails, and so on. Children and adolescents should be monitored or prohibited from engaging in many of these Internet activities. In particular, the online business application tools that parents use should not become the experimental Internet tools for children. The Internet access paths and privileges of adults should be separated from the online Web access paths of children.

Children are the most valuable resources and future leaders in any society. The power of a nation relies upon the strength of its families. The citizens and the government are the main stakeholders in protecting children in cyberspace. Parents, teachers, communities, and governments must forge partnerships and take proactive positions to confront the continued misuse of the Internet by children. The government has never taken the position that parents are inept and incapable of providing guidance to the children in cyberspace. However, the government has taken the position that the filtering technologies are not effective at screening out every inappropriate website. The combination of the Internet access training efforts by parents and teachers with the Web filtering technology and legislation is the solution to eradicating the obscene and child pornography materials in cyberspace.
